# Rare genetic variability in human drug target genes modulates drug response and can guide precision medicine

**DOI:** 10.1126/sciadv.abi6856

**Published:** 2021-09-01

**Authors:** Yitian Zhou, Gabriel Herras Arribas, Ainoleena Turku, Tuuli Jürgenson, Souren Mkrtchian, Kristi Krebs, Yi Wang, Barbora Svobodova, Lili Milani, Gunnar Schulte, Jan Korabecny, Stefano Gastaldello, Volker M. Lauschke

**Affiliations:** 1 Department of Physiology and Pharmacology, Karolinska Institutet, 171 77 Stockholm, Sweden.; 2 Orion Pharma R&D, P.O. Box 65 (Orionintie 1), FI-02101 Espoo, Finland.; 3 Estonian Genome Center, Institute of Genomics, University of Tartu, Tartu, Estonia.; 4 Institute of Molecular and Cell Biology, University of Tartu, Tartu, Estonia.; 5 Pharmaceutical Informatics Institute, College of Pharmaceutical Sciences, Zhejiang University, 310058 Hangzhou, China.; 6 Biomedical Research Centre, University Hospital Hradec Králové, Sokolská 581, 500 05 Hradec Králové, Czech Republic.; 7 Dr. Margarete Fischer-Bosch Institute of Clinical Pharmacology, Stuttgart, Germany.

## Abstract

Interindividual variability in drug response constitutes a major concern in pharmacotherapy. While polymorphisms in genes involved in drug disposition have been extensively studied, drug target variability remains underappreciated. By mapping the genomic variability of all human drug target genes onto high-resolution crystal structures of drug target complexes, we identified 1094 variants localized within 6 Å of drug-binding pockets and directly affecting their geometry, topology, or physicochemical properties. We experimentally show that binding site variants affect pharmacodynamics with marked drug- and variant-specific differences. In addition, we demonstrate that a common *BCHE* variant confers resistance to tacrine and rivastigmine, which can be overcome by the use of derivatives based on squaric acid scaffolds or tryptophan conjugation. These findings underscore the importance of genetic drug target variability and demonstrate that integration of genomic data and structural information can inform personalized drug selection and genetically guided drug development to overcome resistance.

## Teaser

Rare genetic variants in human drug targets affect drug efficacy and can guide personalized drug selection and drug development.

## INTRODUCTION

The response to medications differs drastically between individuals with many patients not experiencing the intended treatment outcomes due to the manifestation of adverse drug reactions (ADRs) or insufficient treatment response. Lack of response constitutes the most common reason for project terminations in drug development, with more than half of all candidate drugs that enter clinical trials failing because of efficacy issues (*1*, *2*). In addition, ethnogeographic or interindividual variability in drug response is also frequently observed for marketed drugs (*3*, *4*). Overall, it is estimated that 10 to 45% of patients do not respond appropriately to pharmacological treatment (*5*), and the fraction of nonresponders can be even higher for certain diseases, such as major depressive disorder where more than half of all patients do not respond to first-line antidepressant therapy (*6*).

Genetic polymorphisms in genes involved in drug disposition, such as drug-metabolizing enzymes and drug transporters, have long been described as biomarkers to predict therapeutic efficacy or ADR risks (*7*, *8*). Well-established examples include *DPYD* genotyping to guide fluorouracil treatment (*9*), *CYP2C19* genotype–guided antiplatelet therapy (*10*), and *CYP2D6* profiling to derive therapeutic recommendations for tamoxifen (*11*). In contrast, how polymorphisms in genes encoding drug targets affect drug response is less studied, at least in part, because of their extensive molecular diversity (*12*, *13*). While the frequencies of loss-of-function variation in human drug targets have been recently mapped (*14*), the extent to which drug target variability affects drug efficacy has not been evaluated.

For a given drug to be effective, the parent molecule or one or more of its metabolites must bind to the target protein and modulate its activity. Genetic variations in drug target genes can affect expression levels or functionality of the gene product, thus altering the stoichiometry of drug molecules to functional target proteins at a given dose. Furthermore, variants that affect amino acids in the drug-binding pocket can directly influence drug-target associations by altering pocket geometry, topology, or physiochemical interactions. Recent seminal work showed that naturally occurring missense variants in drug-binding sites within genes encoding members of the G protein–coupled receptor (GPCR) family of drug targets constitute important determinants of drug response whose consideration could increase prescription precision and reduce socioeconomic costs by up to 500 million British pounds annually (*15*). However, how drug target variability in genes other than GPCRs might modulate drug response has not yet been investigated.

Here, we systematically profiled the genetic variability of all human protein drug targets based on whole-exome and whole-genome sequencing data from 138,632 unrelated individuals across seven ethnogeographic groups. In total, we identified 479,860 naturally occurring exonic variants, of which 82,884 were predicted to affect gene function using a set of 17 partly orthogonal computational algorithms. By leveraging available high-resolution crystal structures, we identified 1094 variations that affected amino acids within 6 Å of the binding drug molecule. To evaluate the relevance of these findings, we experimentally validated the effects of variants on the response to drugs across diverse classes, including angiotensin-converting enzyme (ACE) inhibitors (ACEis), cholinesterase inhibitors, and microtubule polymerization blockers. Binding site variability significantly affected the activity of ACEis with >10-fold differences in response between tested ACE variants. Similarly, tacrine strongly inhibited reference sequence cholinesterase, whereas the common D98G binding site variant (minor allele frequency = 1.2%) was resistant to tacrine. By using a library of tacrine-squaramide derivatives, we identified compounds with strong inhibitory effects on both reference and mutant enzyme, thus overcoming drug resistance. Combined, these data suggest that drug target variability provides an important determinant of interindividual variability in drug response and that integration of genomic and structural information can provide promising means to guide personalized drug selection to optimize drug response.

## RESULTS

### Overview of the genetic variability profile in human drug targets

**Fig. 1.**
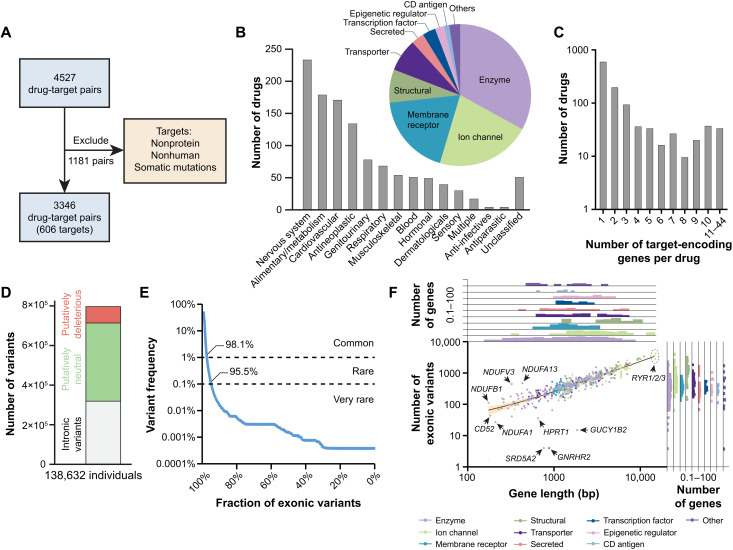
The genetic landscape of human drug targets. (**A**) After exclusion of drugs with nonprotein or nonhuman targets or drugs targeting only specific somatic mutations, we obtained 606 genes encoding the target proteins of 1155 FDA-approved drugs, resulting in a total of 3346 unique drug-target pairs. (**B**) The analyzed drugs were distributed across anatomical therapeutic chemical (ATC) classifications. The most common targets were enzymes and ion channels followed by membrane receptors and structural components. (**C**) Column plot showing the number of target encoding genes per drug. (**D**) Across 138,632 individuals, we identified a total of 798,842 variants of which 479,860 were exonic. Using stringent computational assessments of pathogenicity (see Materials and Methods), 82,884 variants were identified as putatively deleterious. (**E**) The majority of exonic variants in drug target genes are rare with minor allele frequency (MAF) <1%. (**F**) Variant numbers differed >100-fold between drug targets, primarily because of differences in gene length (*R*^2^ = 0.87). Confidence bands (95%) are shown in yellow. The gene density distributions for gene length and variant number are shown across the different protein classes as histograms on top and on the right of the scatter plot, respectively.

To characterize human drug target variability, we first obtained all drug targets (*n* = 893) for 1578 U.S. Food and Drug Administration (FDA)–approved drugs (*16*). We excluded all nonhuman (e.g., directly targeting pathogens; *n* = 129) and nonprotein targets (e.g., targeting DNA or chelating metal ions; *n* = 10) as well as all target variants that only arise because of somatic mutations (*n* = 60), resulting in a total of 606 target proteins targeted by 1155 drugs that give rise to a total of unique 3346 drug-target pairs (Fig. 1A and table S1). The majority of these drugs target the nervous system (*n* = 242; 21%), alimentary tract and metabolic system (*n* = 178; 15%), and cardiovascular system (*n* = 171; 15%), whereas only few anti-infectives (*n* = 4; 0.3%) and antiparasitic drugs (*n* = 3; 0.3%) had human host cell protein targets (Fig. 1B). Most human drug targets were enzymes (*n* = 204; 34%), ion channels (*n* = 131; 22%), and membrane receptors (*n* = 113; 19%). Notably, 45.7% of all drugs are associated with different target-encoding genes (Fig. 1C). To corroborate the accuracy of these annotations, we matched the target annotations by Santos *et al*. (*16*) against the Therapeutic Target Database (*17*). Notably, we find good overlap between both resources with 86.2% of drugs being annotated with the same target in both databases, albeit sometimes different isoforms (table S2). However, for some drugs, particularly those targeting the central nervous system, the exact mechanism of action is not firmly established.

Next, we analyzed the genetic variability in all 606 human drug target genes. Using sequencing data from 138,632 individuals, we identified a total of 798,842 variants, of which 479,860 were exonic (Fig. 1D). Computational predictions further indicated that 82,884 (24.3%) exonic variants were likely to have impact on gene functions. The vast majority of these variants were rare (98.1%) or very rare (95.9%) with minor allele frequencies <1 and 0.1%, respectively (Fig. 1E). Furthermore, more than half of all exonic variants (*n* = 246,768 variants; 51.4%) were singletons. Target genes that encode structural proteins harbored the highest number of exonic variants (on average, 820 missense variants and 70 nonsense variants per target), whereas genes that encode CD antigens were the least variable drug targets (on average, 159 missense variants and 11 nonsense variants per target; fig. S1). Few proteins were devoid of missense variants, including *SRD5A2* (targeted by dutasteride and finasteride), *GUCY1B2* (various guanylate cyclase modulators), and *GNRHR2* (danazol); no nonsense variants were found in *NDUFA1* (metformin), *PRPS1* (nelarabine), *KCNA4* (dalfampridine and guanidine), *CACNG8* (bepridil), and *CD52* (alemtuzumab).

The majority of drug target genes harbored more than 100 variants with the highest number of variants found in the ryanodine receptor family (*RYR1*, *RYR2*, and *RYR3*; *n* > 2700 variants each; Fig. 1F). The calcium channels RyR1 and RyR3 (encoded by *RYR1* and *RYR3*) are targeted by the muscle relaxant dantrolene, whereas RyR2 (*RYR2*) is targeted by the vasodilator hydralazine. In contrast, only five variants were found in *SRD5A2* and *GNRHR2*, which encode the targets of the antiandrogens dutasteride and finasteride and the antiestrogen danazol, respectively.

While the number of variants correlated overall well with gene length [coefficient of determination (*R*^2^) = 0.87], 27 targets differed more than twofold from the average variability (Fig. 1F). Even within closely related targets, the extent of genetic variability could differ extensively. For instance, 444 and 473 variants were identified in the NADH (reduced form of nicotinamide adenine dinucleotide) dehydrogenase complex components *NDUFV3* [1.4 variants per kilo–base pair (kbp)] and *NDUFA13* (1.1 variants/kbp), whereas *NDUFA1* only harbored 27 variants (0.1 variants/kbp).

### Systematic analysis of drug-binding site variability

We hypothesized that variations localized directly within the drug-binding pockets might be most likely to affect drug action by affecting pocket geometry or topology or by altering the physiochemical interactions between drug and target, which might affect the thermodynamic stability of the protein-ligand complex. To estimate the impact of genetically encoded drug-binding site variability, we mapped all variants onto the corresponding three-dimensional protein structures of the respective drug target. To this end, we first extracted all drug targets for which high-resolution crystal structures of the drug bound to its human target were available or could be modeled with high confidence (see Materials and Methods for details), thus assuring exact drug- and target-specific binding site information (table S3).

**Fig. 2.**
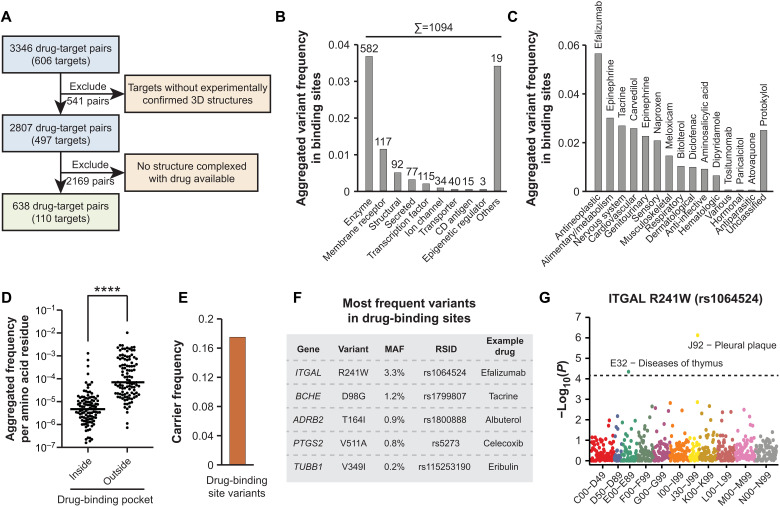
Characterization of the genetic variability in drug-binding sites. Only variants that affected an amino acid within 6 Å of the bound drug as determined by crystallographic data are considered. (**A**) High-quality structural information of the target protein complexed with the respective drug was available for 110 of 606 targets, corresponding to 638 drug-target pairs. (**B**) The aggregated variant frequency in binding sites is shown for the different target categories. The number of binding site variants is indicated on top of the respective columns. Note that drug-binding sites in enzymes were most variable (*n* = 582), whereas only few variants (*n* = 34) were identified in the binding sites of drugs targeting ion channels despite being similarly common drug targets (compare Fig. 1B). (**C**) Drug-binding site variants were most common in targets of antineoplastic drugs and drugs targeting the alimentary system, whereas variants affecting hormonal and antiparasitic medications targeting the host were very rare. Drugs targeting the most variable binding site in each category are indicated. (**D**) Comparison of aggregated variant frequency within and outside of drug-binding sites. (**E**) Overall, approximately one in six individuals (aggregated variant frequency = 17.5%) carries at least one drug-binding site variant. (**F**) The most frequent binding site variants and their corresponding drugs are shown. MAF, minor allele frequency; RSID, rs number. (**G**) For the most common variant rs1064524 in *ITGAL*, a PheWAS in the Estonian population identified associations with two biologically plausible phenotypes with phenome-wide significance. ATC codes are shown on the abscissa and significance as −log(*P*) on the ordinate. Dashed line indicates phenome-wide significance threshold after Bonferroni correction. Other variants were too rare for PheWAS analyses.

In total, such high-quality structural information of targets complexed with approved drugs was available for 638 drug-target pairs (486 drugs and 110 targets; Fig. 2A). Across these targets, 1094 naturally occurring genetic variations resulted in amino acid exchanges in close proximity (≤6 Å) of the bound drug (table S4). Alanine to threonine (A > T, 2.5%) or valine (A > V, 2.5%) as well as arginine to cysteine (R > C, 2.7%) or histidine (R > H, 2.7%) were the most common substitution types (fig. S2). In contrast, the inverse amino acid exchanges were less common with the most notable differences being observed for cysteine to arginine, which was >10-fold less abundant (C > R, 0.2%). Overall, most variants were identified in enzyme-binding sites with an aggregated allele frequency of 3.6% (*n* = 582), whereas the variability in the binding pockets of CD antigens (*n* = 15) and epigenetic regulators (*n* = 3) was very low (aggregated allele frequency < 0.1%; Fig. 2B). While ion channels were the second most common drug target class, the total number (*n* = 34) and the aggregated frequency (<0.1%) of variants in the binding sites of drugs targeting in ion channels were very low. Most binding site variants affected targets of antineoplastic and immunomodulating agents (aggregated allele frequency = 5.6%), whereas only few variants were found in the binding pockets of proteins targeted by antiparasitic drugs or hormonal modulators (aggregated allele frequency < 0.1%; Fig. 2C). Across all genes variability in binding sites was substantially lower than outside of binding pockets (*P* < 0.0001, paired *t* test), suggesting that drug-binding sites are enriched in functionally important domains (Fig. 2D). Furthermore, variability was slightly but significantly lower in the binding pocket of small molecules compared to the epitopes recognized by therapeutic antibodies (fig. S3). In total, the carrier frequency of all 1094 binding site variants was 17.5%, suggesting that approximately one in six individuals carries at least one missense variant in the binding sites, potentially affecting the binding of at least one FDA-approved medication (Fig. 2E).

The most frequent binding site variants were R241W in *ITGAL* (rs1064524), D98G in *BCHE* (rs1799807), and T164I in *ADRB2* (rs1800888) within the binding pockets of efalizumab, tacrine, and albuterol, respectively (Fig. 2F). To evaluate whether these variants might manifest in phenotypes, we conducted a phenome-wide association study (PheWAS) analysis using genetic data linked with longitudinal electronic medical records from 51,138 individuals of the Estonian Biobank [Fig. 2G and (*18*)]. Notably, we found that rs1064524 in *ITGAL* [minor allele frequency (MAF)_Biobank_ = 4.3%] associated with phenome-wide significance (Bonferroni correction) with pleural plaques (β = 1.9; *P* = 7.5 × 10^−7^) and diseases of the thymus (β = 1.5; *P* = 4.5 × 10^−5^). These results suggest that rs1064524 localizes to a functionally important domain and modulates protein function. By contrast, the other variants were too rare in the Estonian population to allow for reliable PheWAS. Hence, this association provides proof of concept that the integration of structural and genetic data can flag functionally relevant variations, likely due to substantial overlap of drug-binding pockets with active sites or structurally important protein domains.

### Ethnogeographic differences in drug-binding site variability

**Fig. 3.**
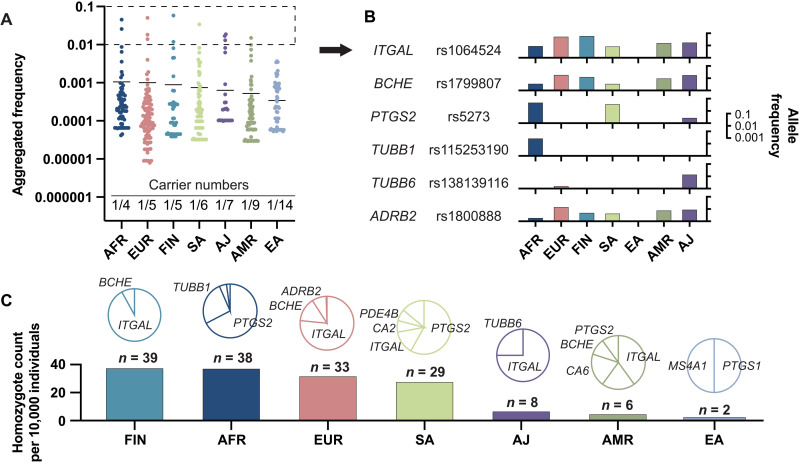
Ethnogeographic variability in human drug-binding sites. (**A**) The aggregated frequencies of binding site variants is shown for every drug target across seven human populations (AFR, African; EUR, European; FIN, Finnish; SA, South Asian; AJ, Ashkenazi Jews; AMR, Latino; and EA, East Asian). The number of binding site variant carriers is shown for each population at the bottom of the plot. (**B**) Interethnic differences in population-specific frequencies are shown for all variants that are common (allele frequency > 1%) in at least one population. Note the considerable differences in population frequencies for most variants. (**C**) The number of homozygous-binding site variant carriers per 10,000 individuals is shown across ethnogeographic groups. Pie charts indicate the genes most commonly affected in the respective population.

Next, we interrogated the genetic variability in drug targets stratified by ethnogeographic groups, as interethnic differences can contribute considerably to differences in pharmacological response. By analyzing drug-binding site variability across seven major human populations, we found that drug-binding site variation differed approximately threefold across groups (Fig. 3A). Variability was highest in individuals of African ancestry where 1 in 4 individuals harbored at least one binding pocket variant and lowest in East Asian population with one variant per 14 individuals.

Among the most abundant variants, large ethnogeographic differences were observed. While rs1064524 in *ITGAL* and rs1799807 in *BCHE* were relatively common in all populations studied except East Asians, rs115253190 in *TUBB1* and rs138139116 in *TUBB6* were almost specific to African populations and Ashkenazim, respectively (Fig. 3B). Overall, the highest number of homozygote-binding site variant carriers was found in the Finnish population (Fig. 3C). Here, 39 per 10,000 individuals were homozygous for binding pocket variants in *ITGAL* and *BCHE*. In contrast, the rates of homozygotes in Ashkenazi Jews, Latinos, and East Asians were much lower with 8, 6, and 2 per 10,000 individuals, respectively (Fig. 3C). These results demonstrate that binding site variability signatures are distinctly different across populations and could contribute to ethnogeographic differences in drug response.

### Binding site variants affect drug pharmacodynamics

Heterologous expression of reference and variant genes coupled to functional assays using appropriate end points is considered as the gold standard to evaluate the functional impact of pharmacogenetic variants (*19*, *20*). To elucidate the functional effect of binding site variants, we experimentally evaluated the effect of binding site variants in targets from diverse therapeutic areas (cardiology, oncology, and neurology). Specifically, we tested five *ACE* variants in the binding pocket for ACEis, which most guidelines recommend as the first-line treatment for the management of hypertension, seven tubulin variants (*TUBB1*) in the binding site of the polymerization inhibitor eribulin, and seven variants in the cholinesterase (*BCHE*)–binding site for the anti–Alzheimer’s disease (AD) medications tacrine and rivastigmine.

**Fig. 4.**
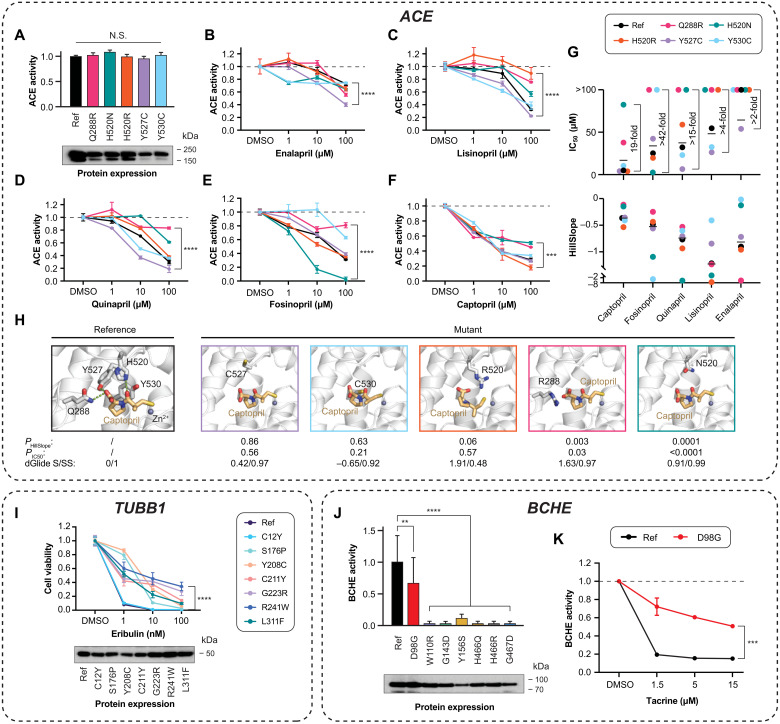
Naturally occurring drug-binding site variability affects drug response in vitro. Effects of binding site variants in *ACE* (**A** to **H**), *TUBB1* (**I**), and *BCHE* (**J** and **K**) were evaluated using functional assays (see Materials and Methods). (A) None of the *ACE* drug-binding site variants had major impacts on ACE expression or baseline ACE activity. Data are shown as means ± SEM; *n* = 3. Dose-response curves of reference ACE and its variants are shown to the clinically approved ACEis enalapril (B), lisinopril (C), quinapril (D), fosinopril (E), and captopril (F). ***P < 0.001; ****P < 0 (*F* test). (G) The IC_50_ values and HillSlope coefficients are shown for each ACE variant–ACEi pair, and the largest fold change across variants is indicated for each drug. (H) Cocrystallized structure of captopril binding to ACE [Protein Data Bank (PDB) ID: 1UZF] and docking poses of captopril with the five ACE variant structures are shown. Atom color code in sticks: oxygen (red), nitrogen (blue), and sulfur (yellow). The zinc ion and hydrogen bonds are shown as violet sphere and green dashed lines, respectively. Parameters indicating the differences between wild-type and variants are shown below the structures. (I) Eribulin effect in cells transfected with reference *TUBB1* or naturally occurring *TUBB1* variants. Note that viability in variant carriers is strongly increased indicating eribulin resistance. (J) With the exception of D98G, drug-binding site variants in *BCHE*-abrogated enzymatic function (**P < 0.01; ****P < 0.0001; heteroscedastic two-tailed *t* test; *n* = 3). (K) Inhibitory effects of the cholinesterase inhibitor tacrine were strongly reduced in D98G compared to reference enzyme (***P < 0.001; *F* test). N.S., not significant; DMSO, dimethyl sulfoxide.

Using a recently developed live cell assay based on aggregation-induced emission in which ACE catalyzes the enzymatic cleavage of the nonfluorescent peptide probe tetraphenylethene (TPE)–Ser-Asp-Lys-Pro (SDKP) into fluorescent TPE (*21*), we first evaluated the baseline activity of ACE and its variants and found no significant differences (Fig. 4A). Subsequently, we evaluated the inhibitory effects of the commonly used ACEis—captopril, enalapril, lisinopril, quinapril, and fosinopril. Notably, we observed drastic drug- and variant-specific differences (Fig. 4, B to F). The largest variability in drug response was found in fosinopril, which inhibited only 19 ± 4% SEM of Q288R, 69 ± 1% of the reference enzyme, and 97 ± 3% of the H520N variant. Similarly, large differences were observed for lisinopril (inhibition between 78 ± 1% of Y527C and 11 ± 9% of H520R) and quinapril (inhibition between 81 ± 6% of Y527C and 17 ± 2% of Q288R).

Y527C (average inhibition, 71 ± 4%) and the reference enzyme (average inhibition, 61 ± 9%) were overall most sensitive to ACEi inhibition, whereas Q288R was most resistant (average inhibition, 32 ± 8%). Captopril was the most potent inhibitor of the reference enzyme and of H520R, Y527C, and Y530C variants, inhibiting 72 ± 2% (reference enzyme), 82 ± 3%, 73 ± 2%, and 66 ± 1% of ACE activity, respectively [median inhibitory concentration (IC_50_) = 4.2 to 8.6 μM, HillSlope: −0.5 to −0.4], whereas fosinopril (97 ± 3% inhibition) and quinapril (81 ± 6% inhibition) were most effective for H520N (IC_50_ = 2.4 μM, HillSlope = −1.1) and Y527C (IC_50_ = 6.5 μM, HillSlope = −0.7), respectively (Fig. 4G). Enalapril constituted the least effective ACEi, inhibiting only 26 to 60% of ACE activity across reference and variant ACEs (IC_50_ > 50 μM; Fig. 4G). Docking analyses showed that for captopril as the most potent ACEi, Y527C and Y530C, which did not significantly affect captopril inhibition (*P*_HillSlope_ and *P*_IC50_ > 0.2), had only minor impacts on ligand interaction stability and shape similarity (Fig. 4H). In contrast, docking indicated larger effects on binding stability for Q288R and H520N that significantly reduced sensitivity to captopril (*P*_HillSlope_ and *P*_IC50_ < 0.05).

In addition, we experimentally tested the sensitivity of seven variants in *TUBB1*, encoding tubulin β1, a common target for chemotherapeutic drugs that act by disrupting mitotic spindle dynamics (*22*) on the cytotoxicity of the microtubule-destabilizing halichondrin eribulin. While eribulin at 1 nM concentration already reduced viability in cells expressing reference tubulin or C12Y by 92 ± 0.6 and 89 ± 0.5%, respectively, drug effects were significantly reduced in cells expressing the tubulin β1 variants Y208C (14 ± 1%), S176P (21 ± 3%), R241W (40 ± 5%), L311F (48 ± 2%), G223R (55 ± 3%), and C211Y (58 ± 4%), suggesting partial resistance to eribulin action (Fig. 4I).

Last, we tested the inhibitory effect of tacrine on seven variants in its BCHE-binding pocket. Notably, six of these variants abrogated enzymatic activity and were thus not tested for their effect on tacrine pharmacodynamics (Fig. 4J). The remaining variant D98G reduced BCHE baseline activity by 51 ± 41% and conferred strong tacrine resistance (tacrine IC_50_ for D98G >15 μM compared to <1 μM for the reference enzyme; Fig. 4K). Combined, these data reveal drastic differences in drug sensitivity between naturally occurring variants across therapeutic areas and target classes and demonstrate that consideration of genetic drug target variability in drug selection can optimize personalized pharmacodynamics.

### Genetically guided selection of pharmacological alternatives can overcome drug resistance in vitro

**Fig. 5.**
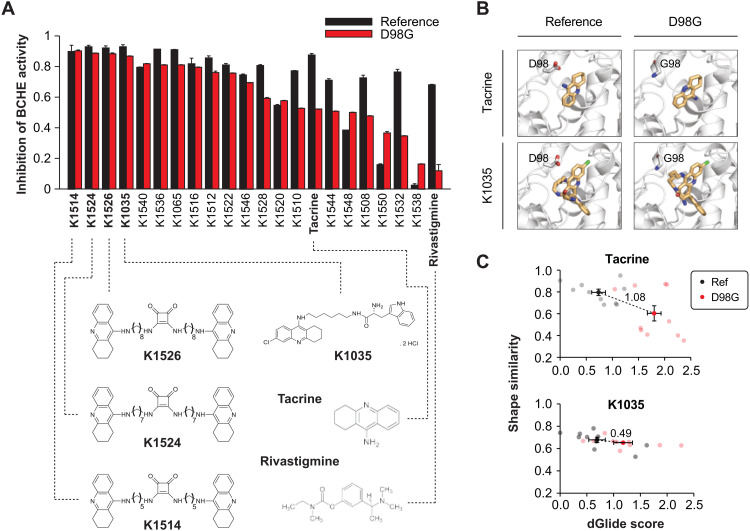
Resistance caused to drug-binding site variability can be overcome using drug derivatives. (**A**) Inhibitory effect on reference and D98G BCHE were evaluated for the cholinesterase inhibitors tacrine and rivastigmine as well as 20 tacrine derivatives. Data are shown as means ± SEM; *n* = 4. Chemical structures of the most potent tacrine derivatives (K1504, K1524, K1526, and K1035) are shown below. Structures of tacrine and rivastigmine are shown for reference. (**B**) Matrix representation of reference and D98G BCHE structures interacting with tacrine (PDB ID: 4BDS) and K1035 (PDB ID: 6I0C). Note that while the positioning of tacrine is strongly altered in D98G, K1035 poses remain invariant. (**C**) Shape similarity and docking score (indicated by dGlide score) of the docked ligand poses in D98G compared to the cocrystallized ligands in (B). Note that the divergence of reference and variant structures (indicated by dashed lines) are considerably higher for tacrine than for K1035. Error bars represent SEM of the 10 best ligand-protein poses for both reference and D98G BCHE.

Next, we investigated whether drug resistance could be overcome using structurally related derivates to pave the way for genetically guided drug development for otherwise resistant drug targets. To this end, we focused on BCHE_D98G_ because of its high frequency (MAF = 1.2%). First, we tested the inhibitory effect of the two approved nonselective cholinesterase inhibitors, tacrine and rivastigmine, and found that while both drugs inhibited reference BCHE by 87 and 68%, respectively, their inhibitory effect was drastically reduced for BCHE_D98G_ (52% for tacrine and 12% for rivastigmine; Fig. 5A). We thus tested a panel of 20 tacrine, 6-chlorotacrine, or 7-methoxytacrine derivates using squaramide as a structural scaffold [table S5 and (*23*)]. Notably, three compounds (K1514, K1524, and K1526) that share a similar homodimer structure with methylene linker lengths between five and eight carbons inhibited both the reference and D98G variant enzyme with equal potency (Fig. 5A). Similarly, K1035, a chlorotacrine-tryptophan heterodimer previously shown to inhibit BCHE at low nanomolar concentrations (*24*), strongly inhibited both reference and D98G variant. Notably, the promising tacrine derivatives are predicted to be central nervous system available by parallel artificial membrane permeation assays (*25*) and have shown strong potential to be centrally active (*24*).

To analyze the molecular details of these observations, we conducted structural analyses using K1035 and tacrine for which experimentally determined crystal structures with the reference enzyme were available. Docking studies of tacrine to reference and D98G BCHE revealed substantial steric flexibility, resulting in a 180° reorientation of the drug molecule in the pocket of the D98G variant (Fig. 5B). In contrast, the configuration of K1035 was not majorly altered between reference and variant, and its shape similarity to the cocrystallized ligand pose remained largely unaffected by the D98G variant (Fig. 5C). These results demonstrate that structural derivation using squaramide motifs or other scaffolds can overcome variant-mediated drug resistance, thus opening previously unidentified avenues for genetically guided drug development specifically targeted at resistant variants.

## DISCUSSION

While genetic variability in genes involved in drug disposition is included in the labels of >130 drugs, drug target variations are only included in the labels of warfarin (targeting VKORC1), cystic fibrosis transmembrane conductance regulator–targeting medications, such as ivacaftor and lumacaftor, and drugs targeting specific somatic mutations in oncology. These differences might be explained, at least in part, because of the larger heterogeneity of drug targets compared to genes involved in drug transport and metabolism, which complicate the experimental interrogation of pharmacodynamic variants. Few studies have systematically evaluated the genetic variability in drug targets and found that the genetic variability in drug-related genes is extensive with a multitude of variants predicted to result in functional consequences (*26*–*29*). However, systematic investigations using structural mapping approaches of population-scale genomic data across approved medications and their targets have not been performed. We here present a comprehensive map of naturally occurring drug-binding site variability of all human drug targets with available experimentally derived high-resolution crystal structures based on genomic information from 138,632 individuals, covering 42% of all approved drugs.

We find that approximately one in six individuals carries at least one variant within a drug-binding pocket. However, as not all targets had structural binding information, the true total frequency of binding site variant carriers can be expected to be even higher. To evaluate the functional consequences of genetic variations, a plethora of computational algorithms that base their predictions on a multitude of features, including sequence conservation, structural information, and functional genomic data have been developed (*30*, *31*). While these tools are considered reasonably accurate in estimating variant pathogenicity, i.e., variant effects on endogenous functions, they are not equipped to predict variant effects on drug action. Here, we showed that although the binding sites of drugs and endogenous substrates commonly overlap, computational algorithms showed only minor overrepresentations in the fraction of putatively deleterious binding site variants compared to variants outside of binding pockets for most genes (fig. S4). Furthermore, while variant effect predictors provided overall reliable predictions for the assessment of variant pathogenicity with the best algorithm showing 98% sensitivity and 92% specificity, predictive accuracy drops <76% for variants that were experimentally shown to alter drug pharmacodynamics (fig. S5 and tables S6 and S7). In contrast, PheWAS analysis of a common binding site variant in *ITGAL* (rs1064524), encoding integrin α L, revealed strong associations with pleural plaques, dense hyalinized collagen fibers formed in parietal pleura, and diseases of the thymus. *ITGAL* encodes the lymphocyte antigen CD11A, an integrin that has been shown to be necessary for thymus development (*32*) and transmigration of leukocytes across the mesothelium lining the pleura (*33*), thus providing a mechanistic fundament for the genetic association. While sufficiently powered PheWASs could not be performed for other binding site variants because of their lower frequencies, these findings provide proof of concept that the identification of binding site variability can also pinpoint variations important for endogenous functions.

Our experimental results showed that with one exception (C12Y in *TUBB1*), the tested binding site variants either abrogated endogenous functions or affected drug pharmacodynamics (18 of 19; 95%), thus demonstrating the overall relevance of drug-binding site variability. For ACE-binding site variants, we observe >40-fold differences in IC_50_ between marketed ACEis. For instance, ACE_H520N_ was inhibited by fosinopril with an IC_50_ of 2.4 μM, whereas this variant was highly resistant to all other ACEis (IC_50_ > 82.4 μM; Fig. 4G). Similarly, efficacy of quinapril was sixfold higher in cells expressing ACE_Y527C_ compared to cells expressing the reference enzyme. These results strongly suggest that drug target variability might explain part of the interindividual variability in the management of hypertension and incentivize the adoption of genotype-informed drug selection. We want to emphasize that we tested drug effects in heterologous overexpression systems, resulting in in vitro IC_50_ values (average IC_50_ around 40 μM) that are approximately 10-fold higher than the therapeutic exposure levels in vivo (clinical *c*_max_ values of the different ACEis = 0.2 to 6 μM). While these models allow for the comparative analysis of pharmacogenomic effects of different variants in vitro, results are not intended for direct quantitative translation into the in vivo setting.

The vast majority of binding site variants in drug targets are extremely rare. An exception is BCHE_D98G_, which is relatively common, particularly in Europeans (MAF = 1.8%) and Ashkenazi Jews (MAF = 1.7%). This variant was first described as the major determinant of prolonged apnea in response to muscle relaxants (*34*). Here, we experimentally show that this variant also confers resistance to the AD medications tacrine and rivastigmine. Thus, developing compounds that can improve the pharmacological effects for D98G variant carriers might be an important advancement to improve therapeutic outcomes. Here, we demonstrate that four tacrine derivatives featured improved inhibitory effect on BCHE_D98G_. Furthermore, these compounds have previously been demonstrated to also strongly inhibit acetylcholinesterase (ACHE) (*23*). While ACHE constitutes the primary cholinesterase in the cerebral cortex, BCHE activity increases in the hippocampus and temporal cortex with progressing AD and BCHE inhibition correlates with improved cognition (*35*). Given the distinct expression patterns of the two genes in different brain regions of patients with AD, strong inhibition of both cholinesterases constitutes an important goal for AD management (*36*, *37*). These findings suggest that K1035, K1514, K1524, and K1526 might constitute promising tacrine alternatives for further preclinical development, particularly for D98G carriers (>3% of the European population), and demonstrate that drug derivation can provide appealing strategies to optimize drug efficacy for individuals with genetic variable binding sites.

The presented data demonstrate that genetic variability affecting amino acids in drug-binding pockets can have pronounced effects on drug efficacy, but the clinical validation of these findings remains challenging because of the low frequency of individual binding site variants. The variants rs56040400 and rs373359894 in *C5* that both affect arginine-885 are predicted by our analysis to affect the binding of the complement inhibitor eculizumab, which aligns with trial data from 345 Japanese patients with paroxysmal nocturnal hemoglobinuria, in which all 11 nonresponders carried a variant in p.Arg^885^ (*38*). Similarly, our data suggested that carriers of the *ADRB2* variant rs1800888 exhibit altered response to β_2_-adrenergic receptor agonists, which is corroborated by the finding that carriers of this variant exhibit a fivefold lower response to isoproterenol (*39*). Furthermore, previous studies showed a substantial enrichment of rare genetic variants in drug targets in nonresponders to antiseizure medications (*40*, *41*). Furthermore, germline rare variant burden in multidrug resistance transporters was negatively correlated with outcomes in patients with breast cancer undergoing chemotherapy (*42*). Jointly, these and our data incentivize further mechanistic studies, as well as the design of prospective trials that evaluate the added value and cost-effectiveness of genotype-informed drug selection, ideally across indications, drugs, and health care systems. Before these data are available, we suggest that information about binding pocket variability could already flag individuals at increased risk of nonresponse for increased surveillance, particularly in therapeutic areas where timely response is critical, such as oncology.

In summary, this study comprehensively profiled human drug target variability on an unprecedented scale and highlights the importance of drug target variability for personalized medicine. Our results demonstrate that integration of population-scale genomic data and structural target information can flag variants with important effects on drug pharmacodynamics and inform personalized prescribing and targeted drug development for carriers of variants resistant to conventional treatment.

## MATERIALS AND METHODS

### Data sources

Genetic variants of all drug target proteins and their frequencies were derived from whole-genome and whole-exome sequencing data of 138,632 individuals provided by the Genome Aggregation Database from seven ethnogeographic groups (European, African, Latinos, East Asian, South Asian, Ashkenazi Jews, and Finnish) (*43*). Experimentally determined high-resolution crystal structures of target proteins crystallized with the respective drug were obtained from the Protein Data Bank (PDB) (*44*). The putative functional consequences of genetic variants in drug targets were assessed using 17 partly orthogonal computational algorithms (SIFT, PolyPhen-2, LRT, MutationTaster, MutationAssessor, FATHMM, PROVEAN, MetaSVM, MetaLR, M-CAP, REVEL, CADD, FATHMM-MKL, VEST3, DANN, Eigen, and GERP++). Variants were classified as deleterious if >80% of algorithms predicted functional impacts. In addition, all frameshifts, start-lost, and stop-gain variants as well as all variations affecting canonical splice sites were considered as deleterious.

### Drug target–binding site identification

Drug targets were identified on the basis of recent comprehensive maps of the human druggable genome (*16*). In total, 606 human drug target proteins were identified for which the respective drug did not target a specific somatic mutation. The evaluation of variants within drug-binding sites relies on the accurate identification of binding pockets. Thus, we only considered those targets for our evaluations of which (i) the structure of the human protein complexed with the respective drug was available, (ii) the structure of a homologous animal protein complexed with the respective drug was available, (iii) the structure of a paralogous human protein complexed with the respective drug was available, (iv) the structure of the human protein complexed with a drug with the same backbone chemical structure was available, or (v) the structure of a paralogous human protein complexed with a drug with the same backbone chemical structure was available. For those targets for which only homologous or paralogous protein structures were available, structural agreement was evaluated using Phyre2 (*45*). Model accuracy was considered sufficient if confidence was >90% and sequence identity was ≥30%. Binding pockets were defined as all amino acids within 6 Å of the respective drug molecule using PyMOL (version 2.1.1, Schrödinger LLC).

### PheWAS using the Estonian Biobank

A PheWAS was conducted for the most common drug-binding site variant, rs1064524 in *ITGAL*. Less abundant variants had to be excluded because of the low number of carriers and, accordingly, low statistical power in the Estonian Biobank. International Classification of Diseases (ICD)-10 codes were obtained from the electronic health records of the participants with the number of unrelated individuals ranging from 18,601 to 51,138, depending on the phenotype. PheWAS codes were first grouped into three-character identifiers, and only PheWAS codes C00-N99 (excluding D78, F70-F89, H, and J00-J22) were selected for further analyses. PheWAS codes with less than five cases in total were excluded, resulting in a total of 725 PheWAS codes. PheWAS analysis was performed using the R package PheWAS (*46*) using logistic regression models with gender, birth year, and the first five principle components as covariates. Bonferroni correction was used to account for multiple testing with a *P* value significance threshold of 0.05/725 ≈6.9 × 10^−5^ (where 725 is the number of PheWAS codes analyzed).

### Molecular docking studies

Cocrystallized structure of captopril with ACE [PDB identification (ID): 1UZF; (*47*)], tacrine with BCHE [4BDS; (*48*)], and its derivate K1035 with BCHE [6I0C; (*24*)] were obtained from the Research Collaboratory for Structural Bioinformatics PDB database. To prepare these structures for the molecular docking, we used the Schrödinger Maestro molecular modeling platform (release, January 2019, Schrödinger, LLC, New York, NY) and PyMOL software. The protein-ligand complexes were first refined in Maestro using Protein Preparation Wizard, and the ligand-binding sites were optimized with Prime using a 7-Å threshold. After these refinement steps, the ligands were removed, and selected point mutations were introduced to each protein structure with the Mutagenesis Wizard in PyMOL.

The cocrystallized ligands were docked back to the corresponding wild-type and mutated protein structures to a box located and sized on the basis of the location and size of the ligand in the original (optimized) complex using Schrödinger’s Glide software. Standard precision (SP) parameters were modified by enhancing the planarity of the conjugated *P* groups. The postdocking minimization was conducted for 20 best ligand poses, and the final output was set to 10 best poses per each ligand-protein pair. As binding of captopril to ACE (PDB ID: 1UZF) involves a metal coordination, an additional location restraint for the coordinating thiol group was added to the docking grid to enhance the enrichment of the biologically meaningful docking poses. The *g* score of each pose was compared to the best-scoring wild-type pose of the corresponding drug-receptor complex, and the dGlide score was calculated as Glide *g* score_best-scoring wild-type pose_ – Glide *g* score_studied pose_. For shape and location analysis, we used ShaEP software (*49*) using “onlyshape” and “noOptimization” parameters; the docking poses of each ligand were compared to the shape and binding location of the cocrystallized ligand at the corresponding crystal structure. All docking poses are provided as data file S1 in mol2 format.

### Experimental evaluation of variants in *ACE*, *BCHE*, and *TUBB1*

#### Site-directed mutagenesis

Reference ACE, BCHE, and TUBB open reading frames were inserted into eukaryotic c-Myc–tagged plasmids, and drug target variants were generated using the QuikChange II Site-Directed Mutagenesis Kit (200524, Agilent Technologies AH Diagnostic). The sequences of the primers used to generate the variant proteins are listed in table S8. Following mutagenesis, variant constructs were sequenced to confirm the correct nucleotide mutations.

#### Functional assays

Human embryonic kidney 293 cells were grown in a humidified atmosphere at 37°C and 5% CO_2_ in in Dulbecco’s modified Eagle’s medium (DMEM) without phenol red supplemented with 10% heat-inactivated fetal bovine serum and penicillin and streptomycin (100 IU/ml). Cells were transiently transfected with the respective plasmids using Viromer RED (Lipocalyx, Germany). Expression levels of protein variants were analyzed by Western blots using antibodies against c-Myc (9E10, Santa Cruz Biotechnology).

ACE activity was measured as previously reported (*21*). Cells were seeded in a 96-well plate and transfected with the corresponding ACE reference and variant plasmids. After 24 hours, the medium was replaced with fresh medium containing ACEi (captopril, enalapril, lisinopril, quinapril, and fosinopril) at final concentrations of 1, 10, and 100 μM as indicated. Notably, because enalapril, quinapril, and fosinopril are prodrugs, we used their respective active metabolites (enalaprilat, quinaprilat, and fosinoprilat) instead of the parent compounds for exposure. After 4 hours of exposure, the medium was replaced with the peptide probe TPE-SDKP (50 μM) that emits fluorescence upon ACE-dependent cleavage in tris buffer and incubated for 2 hours at 37°C. Subsequently, ZnCl_2_ was added to a final concentration of 3 mM and incubated for another 60 min after which the results were measured.

BCHE functionality was quantified using Ellman’s assay. Transfected cells were seeded in 96-well format, and after 24 hours, the medium was replaced with fresh DMEM without phenol red. After 48 hours, 50 μl of medium containing the secreted cholinesterase proteins was transferred into a new 96-well plate and was incubated with 50 μl of cholinesterase inhibitors (tacrine, rivastigmine, or tacrine-squaramide derivatives) at a final concentration of 1.5 μM for 30 min. Ellman solution (300 μl) was added to each well, and absorbance was measured at 412 nm using a SpectraMax M2 microplate reader from 1 to 5 min.

To evaluate the effect of tubulin variants, cells were seeded for transfection with the corresponding TUBB1 reference and variant plasmids. After 24 hours, the medium was replaced with fresh DMEM without phenol red containing eribulin at a final concentration of 1, 10, or 100 nM. After 72 hours of exposure, adenosine 5′-triphosphate levels were measured using the CellTiter-Glo kit (Promega) to quantify cell viability.
