# Supplementary material for: Rare genetic variability in human drug target genes modulates drug response and can guide precision medicine
Source: Sci Adv. 2021 Sep 1;7(36):eabi6856. doi: 10.1126/sciadv.abi6856 (PMC8442892; doi:10.1126/sciadv.abi6856)

Science Advances
AAAS

Supplementary Materials for

Rare genetic variability in human drug target genes modulates drug response and can guide precision medicine

Yitian Zhou, Gabriel Herras Arribas, Ainoleena Turku, Tuuli Jürgenson, Souren Mkrtchian, Kristi Krebs, Yi Wang, Barbora Svobodova, Lili Milani, Gunnar Schulte, Jan Korabecny, Stefano Gastaldello, Volker M. Lauschke*

*Corresponding author. Email: volker.lauschke@ki.se

Published 1 September 2021, *Sci. Adv.* **7**, eabi6856 (2021)
DOI: 10.1126/sciadv.abi6856

The PDF file includes:

Figs. S1 to S5
Legends for tables S1 to S8
Legend for data file S1

Other Supplementary Material for this manuscript includes the following:

Tables S1 to S8
Data file S1

SUPPLEMENTARY MATERIAL

Fig. S1. Number of drug target variants in different protein classes. Missense, loss-of-function (LOF) and synonymous variants across different protein classes presented in total variants number (**A**) and number of variants per kb gene length (**B**). Dashed lines represent 1st quartile, median and 3rd quartile.

Fig. S2. Heatmap of amino acid substitution rate within drug binding sites. Substitution rate from reference to variant amino acid is indicated from white (low) to red (high).

Fig. S3. Differences in binding site variability between small molecule binding pockets and epitopes targeted by therapeutic antibodies. The variability in binding pockets is 0.0026±0.002 SEM for antibodies compared to 0.0006±0.0002 SEM for small molecules. p-value refers to homoscedastic two-tailed T-test.

Fig. S4. Deleteriousness of variants in drug binding site indicated by computational prediction tools. Each dot represents the fraction of variants predicted to be deleterious inside and outside the drug binding pocket for one gene.

Fig. S5. Comparison of predictive accuracy between pathogenic and pharmacodynamic variants. A, The predictive accuracy of 17 different computational algorithms was analyzed using 463 disease-annotated variants (203 pathogenic and 260 benign variants). Disease

annotations were obtained from ClinVar. **B**, The predictive accuracy of the same 17 algorithms is analyzed for variants that were experimentally shown to alter drug pharmacodynamics (77 variants that altered drug effects and 23 that did not affect drug efficacy *in vitro*). Functional annotations were obtained from the GPCR database (https://gpcrdb.org/).

Table S1. Description of all analyzed drug targets.

Table S2. Overlap of drug target annotations between the curated data by Santos et al. and the Therapeutic Target Database (TTD).

Table S3. Overview of experimentally characterized crystal structures of drugs complexed with their corresponding targets.

Table S4. List of naturally occurring missense variants within drug binding sites.

Table S5. Chemical structures of the utilized synthetic tacrine derivatives.

Table S6. Computational predictions of the effects of pathogenic variants.

Table S7. Computational predictions of the effects of pharmacodynamic variants.

Table S8. Primers for site direct mutagenesis.

Supplementary Data file S1. Docking poses of captopril, tacrine and K1035 to their corresponding targets in both wild-type (wt) and mutant forms.

Supplementary Figure 1

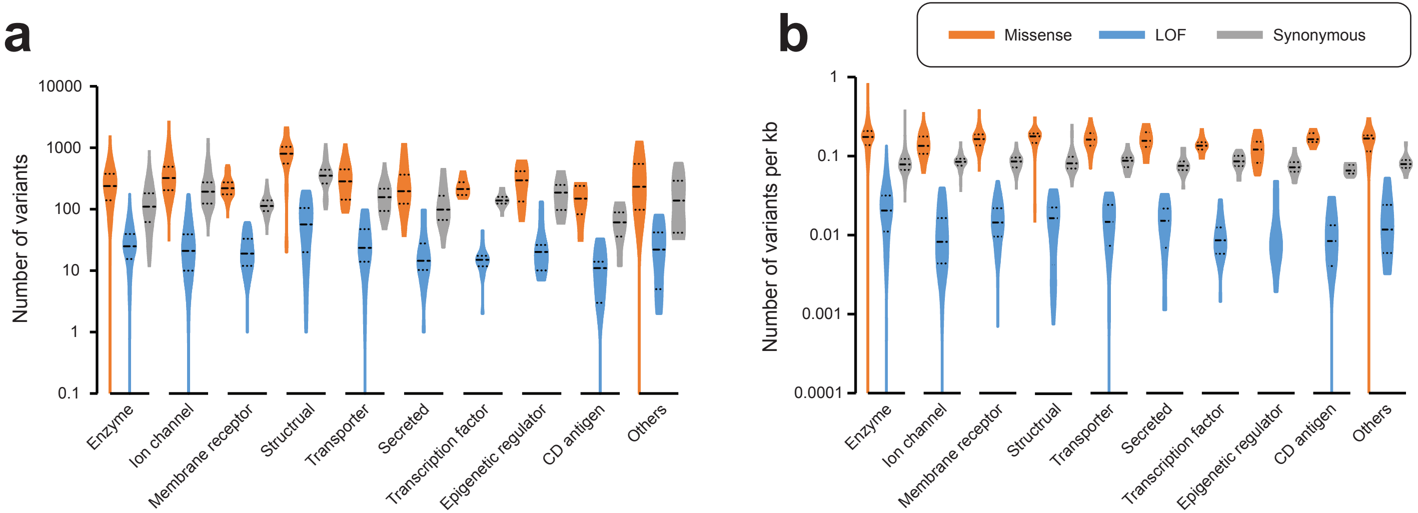

Supplementary Figure 2

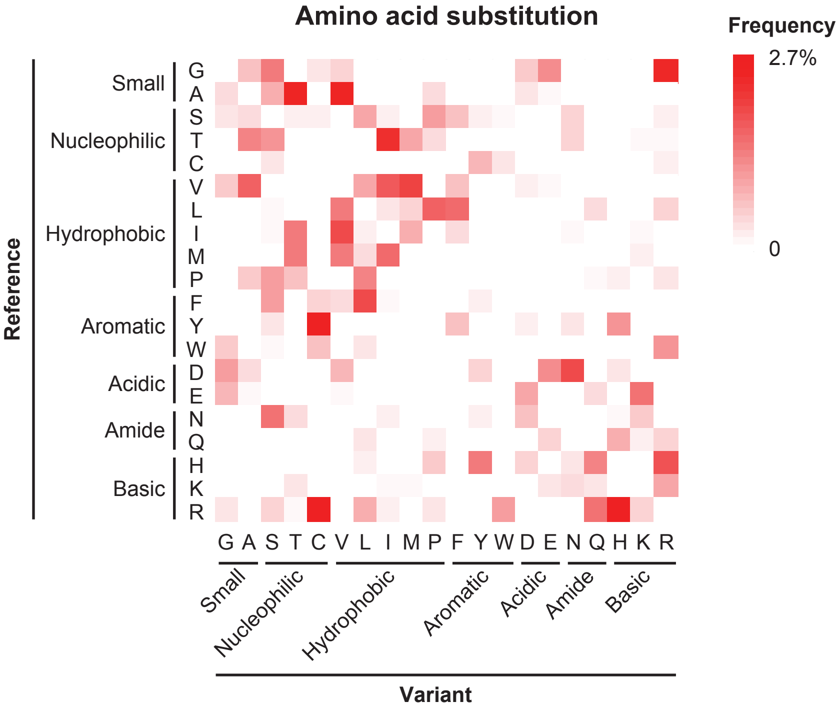

Supplementary Figure 3

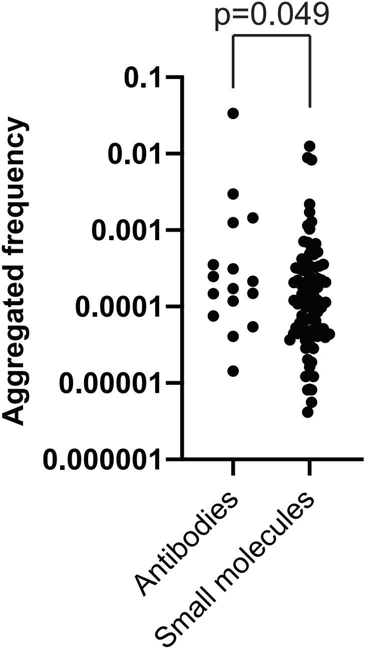

Supplementary Figure 4

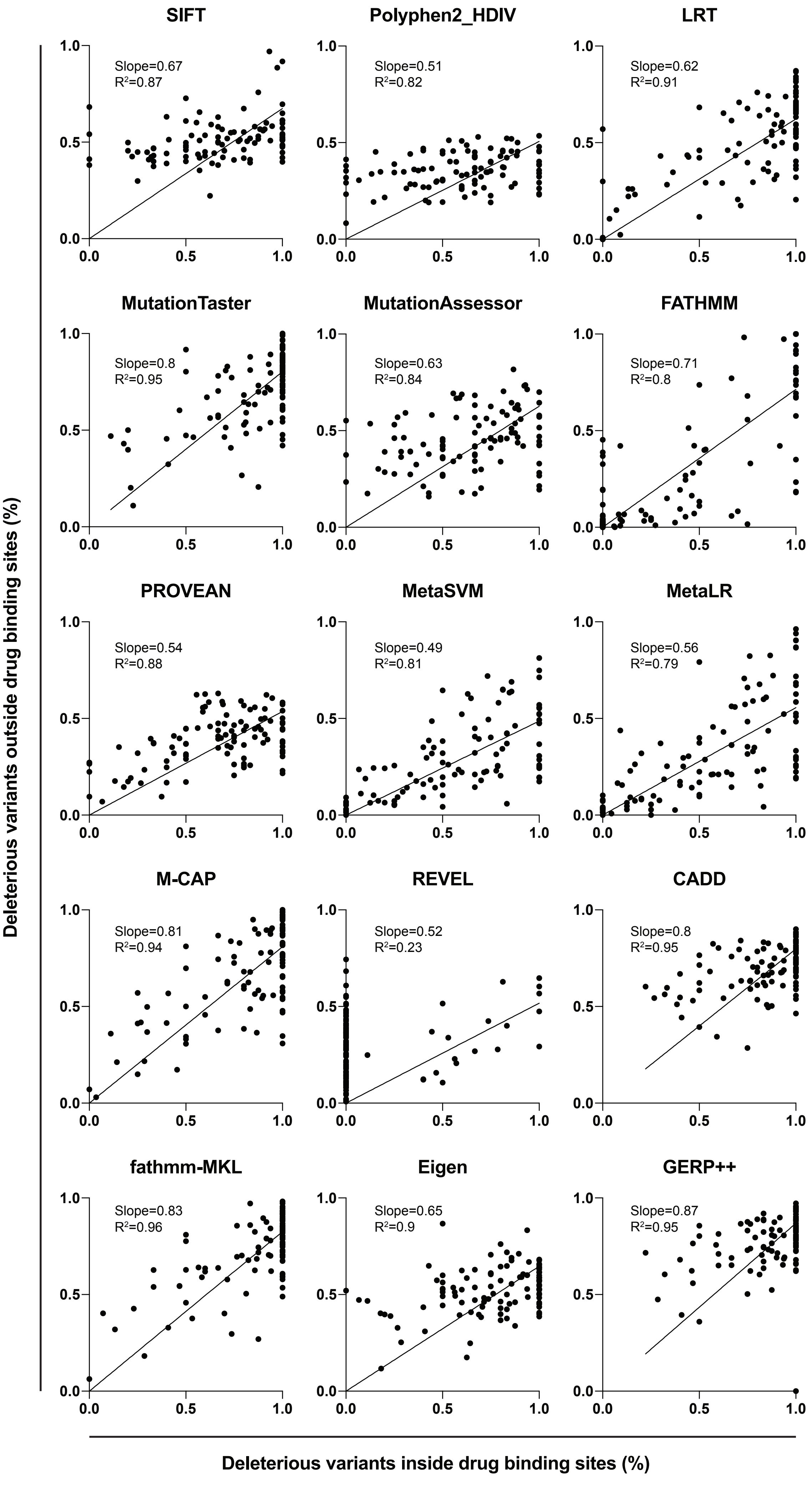

Supplementary Figure 5

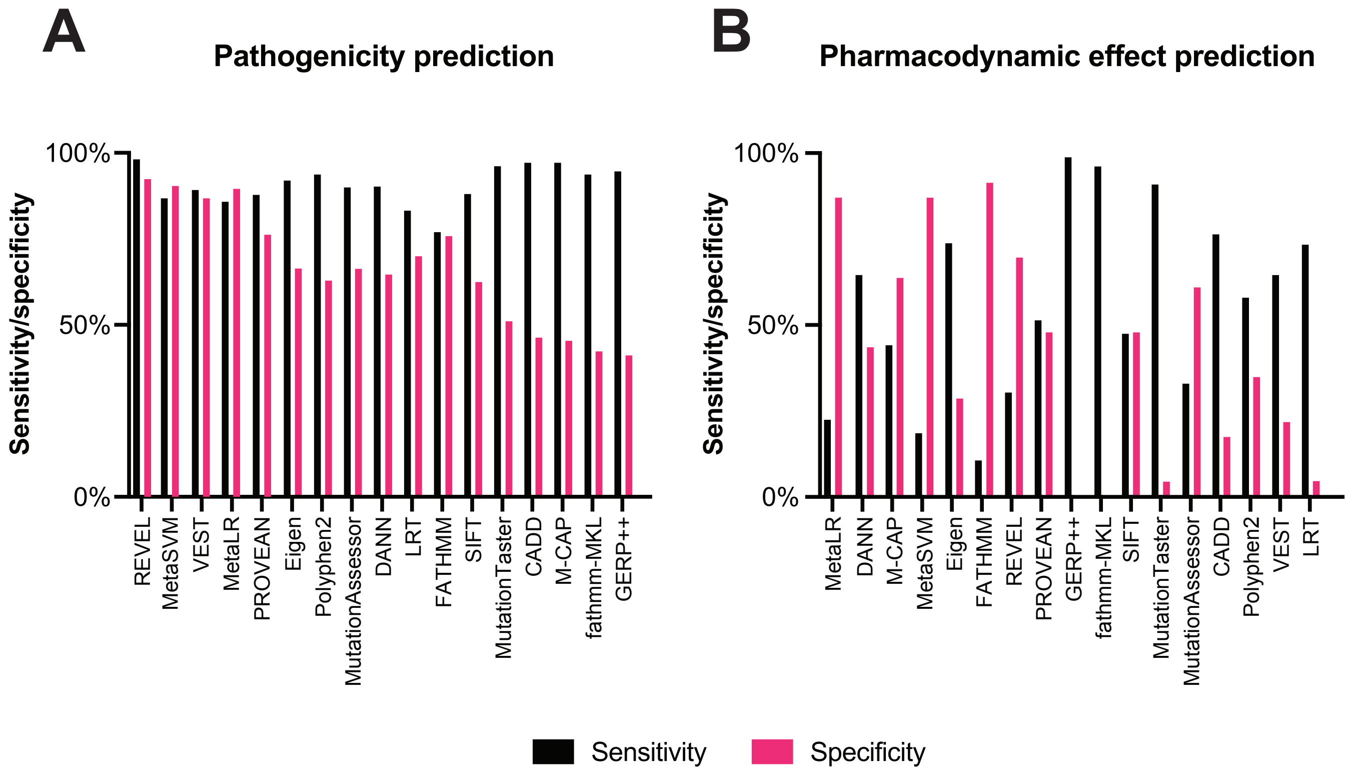

Supplement: Supplementary file 1 — Figs. S1 to S5 Legends for tables S1 to S8 Legend for data file S1 [file sciadv.abi6856_sm.pdf]
